# Co-inhibition of SMAD and MAPK signaling enhances ^124^I uptake in *BRAF*-mutant thyroid cancers

**DOI:** 10.1530/ERC-21-0017

**Published:** 2021-04-23

**Authors:** Kathleen A Luckett, Jennifer R Cracchiolo, Gnana P Krishnamoorthy, Luis Javier Leandro-Garcia, James Nagarajah, Mahesh Saqcena, Rona Lester, Soo Y Im, Zhen Zhao, Scott W Lowe, Elisa de Stanchina, Eric J Sherman, Alan L Ho, Steven D Leach, Jeffrey A Knauf, James A Fagin

**Affiliations:** 1 Human Oncology and Pathogenesis Program, Memorial Sloan Kettering Cancer Center, New York, New York, USA; 2 Department of Surgery, Memorial Sloan Kettering Cancer Center, New York, New York, USA; 3 Cancer Biology and Genetics Program, Memorial Sloan Kettering Cancer Center, New York, New York, USA; 4 Antitumor Assessment Core Facility, Memorial Sloan Kettering Cancer Center, New York, New York, USA; 5 Department of Medicine, Memorial Sloan Kettering Cancer Center, New York, New York, USA; 6 Weill-Cornell Medical College, New York, New York, USA

**Keywords:** thyroid, TGF beta, BRAF, iodide uptake

## Abstract

Constitutive MAPK activation silences genes required for iodide uptake and thyroid hormone biosynthesis in thyroid follicular cells. Accordingly, most *BRAF^V600E^* papillary thyroid cancers (PTC) are refractory to radioiodide (RAI) therapy. MAPK pathway inhibitors rescue thyroid-differentiated properties and RAI responsiveness in mice and patient subsets with BRAF^V600E^-mutant PTC. TGFB1 also impairs thyroid differentiation and has been proposed to mediate the effects of mutant BRAF. We generated a mouse model of BRAF^V600E^-PTC with thyroid-specific knockout of the *Tgfbr1* gene to investigate the role of TGFB1 on thyroid-differentiated gene expression and RAI uptake *in vivo.* Despite appropriate loss of *Tgfbr1*, pSMAD levels remained high, indicating that ligands other than TGFB1 were engaging in this pathway. The activin ligand subunits Inhba and Inhbb were found to be overexpressed in BRAF^V600E^-mutant thyroid cancers. Treatment with follistatin, a potent inhibitor of activin, or vactosertib, which inhibits both TGFBR1 and the activin type I receptor ALK4, induced a profound inhibition of pSMAD in BRAF^V600E^-PTCs. Blocking SMAD signaling alone was insufficient to enhance iodide uptake in the setting of constitutive MAPK activation. However, combination treatment with either follistatin or vactosertib and the MEK inhibitor CKI increased ^124^I uptake compared to CKI alone. In summary, activin family ligands converge to induce pSMAD in Braf-mutant PTCs. Dedifferentiation of BRAF^V600E^-PTCs cannot be ascribed primarily to activation of SMAD. However, targeting TGFβ/activin-induced pSMAD augmented MAPK inhibitor effects on iodine incorporation into BRAF tumor cells, indicating that these two pathways exert interdependent effects on the differentiation state of thyroid cancer cells.

## Introduction

Radioiodine is a key treatment modality for patients with recurrent or metastatic-differentiated thyroid cancer and is also widely applied in the post-operative adjuvant setting. Most papillary thyroid cancers (PTC), which are the most prevalent form of the disease, are associated with clonal non-overlapping activating mutations of genes encoding MAPK pathway signaling effectors, primarily point mutations of *BRAF* and of the three *RAS* genes, as well as fusions of receptor tyrosine kinases (Fagin & Wells 2016). There is an inverse correlation between the MAPK signaling flux of PTCs, as measured by its transcriptional output, and the expression of genes required for iodine uptake and thyroid hormone biosynthesis (Cancer Genome Atlas Research Network 2014). BRAF^V600E^-mutant thyroid cancers have a high MAPK output because this class 1 BRAF-mutant signals as a monomer and is insensitive to the negative feedback effects of ERK on activated RAF dimers (Yao *et al.* 2015). Accordingly, they also have the most profoundly decreased thyroid differentiation score (TDS) (Cancer Genome Atlas Research Network 2014), a quantitative integrated readout of a set of thyroid differentiation markers, and tend to be more refractory to RAI therapy (Xing *et al.* 2005).

Treatment of well-differentiated thyroid cells with TGFB1 impairs TSH-induced expression of thyroid-specific genes such as *Tg* (thyroglobulin) and *Slc5a5* (*Nis*) (Colletta *et al.* 1989). The Santisteban lab showed that pSMAD2/3 binds to the thyroid lineage transcription factor PAX8 and impairs its transactivation of the sodium iodide symporter (*Nis*), and that this is reversed by expression of SMAD7 (Costamagna *et al.* 2004, Riesco-Eizaguirre *et al.* 2009), which prevents SMAD2/3 activation by promoting degradation of the TGFβ receptor (Kavsak *et al.* 2000). Moreover, BRAF^V600E^-induced suppression of Nis expression was shown to be mediated by a TGFB1-driven autocrine loop in PCCL3 (Riesco-Eizaguirre *et al.* 2009). This group also found that the inhibition of *Nis* transcription was MEK-independent, implying that redifferentiation is attainable in the setting of high constitutive MAPK activation. This is inconsistent with the evidence that RAF and MEK inhibitors rescue *NIS* mRNA levels and iodine incorporation in *BRAF*-mutant cell lines, mouse models and patients (Knauf *et al.* 2003, Liu *et al.* 2007, Chakravarty *et al.* 2011, Ho *et al.* 2013, Rothenberg *et al.* 2015, Nagarajah *et al.* 2016). Nevertheless, BRAF^V600E^ clearly increases TGFB1 expression and pSMAD in cell lines and mouse PTCs(Riesco-Eizaguirre *et al.* 2009, Knauf *et al.* 2011),prompting us to investigate the functional role of this pathway in a genetically accurate *in vivo* context.

By using a combination of genetic and pharmacological approaches, we found that pSMAD activation is increased in BRAF-mutant thyroid cancers, and that this is due to promiscuous engagement of activin and TGFβ family ligands with their corresponding receptors. Inhibition of pSMAD activation *in vivo* is insufficient to induce the cancer cells to redifferentiate in the context of constitutive MAPK activation. However, suppression of both MAPK and pSMAD pathways leads to enhancement of radioiodine uptake in cancer cells, an effect that could be leveraged for therapeutic benefit.

## Materials and methods

### Mouse models

Animals used in this study were maintained on a mixed strain background. Animal care and all experimental procedures were approved by the MSKCC Institutional Animal Care and Use Committee. The mouse lines were genotyped by Transnetyx, Inc. (Cordova, TN) using quantitative PCR or by PCR using primers described in Supplementary Table 1 (see section on supplementary materials given at the end of this article).

*TPO-Cre* mice (Kusakabe *et al.* 2004) obtained from Dr Shioko Kimura (NCI) were bred with *LSL-Braf^V600E^* mice (Mercer *et al.* 2005) obtained from Dr Catrin Pritchard (University of Leicester, UK) to generate *TPO-Cre/LsL-Braf^V600E^* (*Braf*) mice, resulting in thyroid-specific knock-in of oncogenic *Braf* (Franco *et al*. 2011).* Tgfbr1*^f/f^ (*TβR1*) mice (Larsson *et al.* 2003), a generous gift from Dr Yosuke Mukoyama (NIH/NHLBI), were bred with *LSL-Braf^V600E^* and *TPO-Cre* to generate *LSL-Braf^V600E^/Tgfbr1^f/f^* and *TPO-Cre/Tgfbr1^f/f^*, respectively. Experimental *TPO-Cre/LSL-Braf^V600E^/Tgfbr1^f/f^* (*Braf/TβR1*) mice were generated by breeding *TPO-Cre/Tgfbr1^f/f^* with *LSL-Braf^V600E^/Tgfbr1^f/f^*.

### Generation of Tre-shTgfbr1 mice

The shRNAs to *Tgfbr1* and *Tgfbr2* were designed using splashRNA (splashrna.mskcc.org) (Pelossof *et al.* 2017) and cloned into the pMSCV retrovirus expression vector. Retroviral particles expressing the shRNAs were generated as previously described (Dow *et al.* 2012) and used to infect a *TPO-Cre/Braf^V600E^ PTC* cell line. Quantitative RT-PCR was used to determine knockdown efficiency and Western blotting for pSMAD to evaluate efficacy for blocking TGFB1-induced SMAD activation (Supplementary Fig. 1D, E and F). *Tgfbr1* shRNA #6, the most effective in blocking TGFB1-induced pSMAD (Supplementary Fig. 1D, E and F), was subcloned into the TGMP vector and used to target *Tre-shTgfbr1* into the Cola1-homing cassette (CHC) in ESCs derived from *TPO-Cre/LSL-Braf^V600E^/RIK/CHC* mice using recombination-mediated cassette exchange (Premsrirut *et al.* 2011, Dow *et al.* 2012). The ESC clones confirmed to have single copy of *Tre-shTgfbr1* targeted to the CHC were microinjected into blastocysts produced from NCI C57BL/6-cBrd/cBrd/Cr (C57BL/6 albino) mice and implanted into CD-1 pseudopregnant mothers enabling the production of chimeric pups. Chimeric pups were bred to generate *TPO-Cre/RIK/Tre-shTgfbr1* mice and experimental *TPO-Cre/Braf^V600E^/RIK/Tre-shTgfbr1* (*Braf/shTβR1*) animals generated by crossing them with *LSL-Braf^V600E^*. Animals were fed doxycycline-impregnated chow (TD01306, Envigo) to induce expression of the *Tgfbr1* shRNA and the appropriate knockdown of *Tgfbr1* was confirmed by quantitative RT-PCR.

### Generation of mouse papillary thyroid cancer cell lines

PTCs from *Braf*, *Braf/TβR1* and *Braf/shTβR1* mice were collected, washed with 1× PBS, minced and placed in digestion medium (MEM with 112 U/mL collagenase type I, 1.2 U/mL dispase and pen/strep). Minced tissues were incubated at 37°C for 45–60 min and then washed twice with Coon’s F12 containing 0.5% bovine brain extract (BBE) (Hammond Cell Tech) and pen/strep/glutamine (PSG) (Gemini Bio Products). Cells were resuspended in Coon’s F12 containing 0.5% BBE and PSG and then plated in CellBind plates (Corning). After culturing for 7–10 days, the culture medium was changed to growth medium (Coon’s F12 containing 5% fetal bovine serum (FBS)(Omega Scientific), 0.5% BBE and PSG). Cells were passaged for at least 1 month and the thyroid cancer origin confirmed by expression of BRAF^V600E^ prior to use.

### Reagents

EW7197 (Vactosertib, Catalog No.S7530) and SD208 (Catalog No.S7530) were purchased from SelleckChem. CH5126766 (CKI) was from Chugai Pharmaceutical Co. Follistatin was purchased from Shenandoah Biotechnologies Inc. The TGFB1 neutralizing antibody 1D11 and its isotype control were obtained from the MSKCC MAB Core.

For *in vivo* experiments, CKI was dissolved in 2 hydroxypropyl-β-cyclo-dextrin and administered once daily by oral gavage at a dose of 3.0 mg/kg. EW7197 was dissolved in artificial gastric fluid formulation (95 mM HCl, 38 mM NaCl and 3.6 mg/mL pepsin) and administered once daily by oral gavage at a dose of 25 mg/kg. Follistatin was dissolved in sterile H_2_O and administered once daily by i.p. injection at a dose of 25 µg/kg. The TGFB1 neutralizing antibody 1D11 and isotype control were suspended in sterile saline and administrated once every 2 days at a dose of 10 mg/kg.

### RNA isolation and quantitative RT-PCR

Thyroid lobes were surgically removed and immediately placed in liquid N_2_. Cell lines were scraped into PBS, washed once with PBS and the cell pellet used for RNA isolation. RNA was isolated using TRIzol (Invitrogen) or RNAeasy (Affymetrix) and 100–500 ng was reverse transcribed with SuperScript III (Invitrogen) in the presence of random hexamers to generate cDNA. Quantitative PCR using the cDNA as a template was done using Power SYBR Green PCR Master Mix (Applied Biosystems) and primer pairs for mouse *β-actin*, *Tgfbr1*, *Tgfbr2*, *Tshr*, *Tg*, *Nis*, *Tpo*, *Pax8*, and *Dusp5* (Supplemental Table 1). The cycle threshold values for β-actin and the target genes were determined with a 7500 real-time PCR instrument (Applied Biosystems) and used to calculate the β-actin normalized relative expression using the QGENE program (Tomas *et al.* 2012).

### ^124^I PET imaging

Imaging was performed using an R4 microPET scanner (Concorde Microsystems) as previously described (Chakravarty *et al.* 2011, Nagarajah *et al.* 2016). Briefly, mice were gavaged with 2.6–3.5 MBq (70–94 μCi) of Na^124^I and were imaged 24 and 72 h later under inhalational isoflurane anesthesia at 1.5 L/min. List-mode data were acquired for 5 min using an energy window of 250–750 keV and a coincidence timing window of 6 ns, histogrammed into 2D projected data by Fourier rebinning, and reconstructed by filtered back-projection using a cut-off frequency equal to the Nyquist frequency. Image visualization and analysis were performed using ASIPro VM software (Concorde Microsystems) by drawing a 3D volume of the target lesions to derive the activity concentrations to the two different time points, which in turn was used to calculate a maximum activity concentration by applying an empirical model as previously described (Jentzen *et al.* 2008). The activity concentration was corrected for partial volume effects using a recovery coefficient derived from previously done phantom studies.

### Immunostaining

Mouse thyroids dissected from surrounding tissues were fixed in 4% paraformaldehyde, embedded in paraffin, sectioned, and stained with hematoxylin and eosin (H&E). Sections were also immunostained for CD45. H&E and IHC for CD45 were performed by the MSK Molecular Cytology Core Facility.

#### Co-immunofluorescent staining for NIS and Pan-cadherin

For immunofluorescence staining, tissue sections deparaffinized and permeabilized with 0.3% Triton were subjected to heat-induced epitope retrieval using citrate-based antigen unmasking solution at pH6 (H-3300; Vector Laboratories). Sections were blocked in PBS containing 3% BSA and 10% normal donkey serum (017-000-121; Jackson Labs) and incubated with rabbit anti-Rat SLC5A5 (kindly provided by Dr Nancy Carrasco, Vanderbilt School of Medicine), followed by A647 labeled donkey anti-rabbit secondary antibodies (A31573; Invitrogen) and sequentially co-stained with A546 labeled pan-cadherin antibody (Sc-515872; Santa Cruz). Z-stack scans of the slides were taken with Pannoramic P250 Flash scanner (3DHistech, Hungary) using 20×/0.8NA objective lens. Regions of interest around the tissues were then drawn and max projection images were exported as .tif files using Caseviewer (3DHistech, Hungary). Images were analyzed using ImageJ/FIJI (NIH, USA) where thresholding and watershedding were used to segment the nuclei in the DAPI channel. NIS expression at the plasma membrane was determined by thresholding the A647/A546 channel and quantifying the co-localization fluorescence intensity at the membrane, and normalizing this to the DAPI/cell counts across the sections.

### Western blotting

Mouse thyroid cancer cell lines were plated in CellBind plates in Coon’s F12 with 5% FBS, 0.5% BBE and PSG and incubated for 24–48 h. Medium was then switched to Coon’s F12 with 0.1% BSA and PSG and incubated for 24–48 h. Cells were then washed with ice-cold PBS and harvested by scraping and centrifugation (1000 ***g*** for 4 min at 4°C). The cell pellets were resuspended in a lysis buffer consisting of 10 mM Tris-HCl (pH 7.5), 5 mM ETDA, 4 mM EGTA, 1% Triton ×100, protease inhibitor cocktail (Sigma) and phosphatase inhibitor cocktail I and II (Sigma). Cells were placed on ice for 10 min and lysed by passing through a p200 tip. Cell debris was removed by centrifugation (18,000 ***g*** for 15 min at 4°C) and the supernatant was collected. Frozen tissues were placed in lysis buffer and then ground with a Polytron homogenizer. Lysates were centrifuged to removed debris (18,000 ***g*** for 15 min at 4°C) and supernatant was collected. Protein concentration for all lysates was determined using the MicroBCA kit (Thermo Fisher Scientific). Ten to fifty micrograms of protein lysate was subjected to SDS-PAGE and transferred to PVDF membranes (VWR). The membranes were probed with the indicated antibody and the target protein detected by incubating with species-specific horseradish peroxidase conjugated IgG’s (Santa Cruz) or IRDye fluorophores and then with ECL reagent (Amersham Biosciences). HRP probed blots were developed using the ECL reagent (Amersham Biosciences) and the signal captured using X-ray films or with the KwikQuantTM Imager (http://kindlebio.com/index.php). IRDye-probed blots were imaged using the LICOR Odyssey imaging system (Licor Biosciences). The following antibodies were used: from Cell Signaling pERK (9101), ERK (9102), pSMAD2^S465/467^ (3101S) and Vinculin (13901S), from Sigma β-actin (A2228), from Santa Cruz SMAD2/3 (sc-8332), from Thermofisher pSMAD2^S465/467^ (44-244G), from Abcam pSMAD^T8^; from Novus NOX4 (NB110-58849). ImageJ was used to quantify band density.

### Human clinical trials

The trials NCT02456701, NCT01947023 and NCT02145143 were performed after approval by the MSKCC Institutional Review Board (IRB numbers 15-046, 13-061 and 14-031, respectively). Written informed consent was obtained from all patients in these studies. Patients received either vemurafenib 960 mg or dabrafenib 150 mg orally twice daily and had sequential biopsies of the same index lesion at baseline (prior to drug) and at 2 weeks while on either vemurafenib or dabrafenib. RNAseq was performed on all samples as described (Dunn *et al.* 2019).

### Datasets and TGFβ family ligand score (TLS)

NCBI GEO human datasets GSE29265, GSE33630 (Dom *et al.* 2012, Tomas *et al.* 2012) and GSE65144 (von Roemeling *et al.* 2015) were downloaded and imported into Partek Genomic suites for normalization and expression analysis. Normalized expression data derived from RNAseq of PTCs in the THCA TCGA dataset (Cancer Genome Atlas Research Network 2014) were obtained from the archive at GDAC Firehose: http://gdac.broadinstitute.org/runs/stddata__2016_01_28/data/THCA/20160128/gdac.broadinstitute.org_THCA.Merge_rnaseqv2__illuminahiseq_rnaseqv2__unc_edu__Level_3__RSEM_genes_normalized__data.Level_3.2016012800.0.0.tar.gz. TGFβ family ligand score is the average log2 fold change of *Tgfb1*, *Tgfb2*, *Tgfb3*, *Inhba* and *Inhbb* compared with the median of all samples. The TGFβ transcriptional output score was determined by calculating the average fold change for all mRNAs in the gene set. The TGFβ gene set consisted of genes found regulated by TGFβ (Coulouarn *et al.* 2008) that were also upregulated in mouse BRAF^V600E^-PTCs compared to normal thyroid and included the following mRNAs: *GNG13, HMGA2, MOSPD3, PCDH1, PDLIM7, PRG4, TGFBR1, TNK2, UBR2* and *ZSWIM4*.

### Statistical analysis

The statistical software GraphPad-Prism (version 8.0; GraphPad Software, Inc.) was used to analyze the data, compute the Pearson correlation coefficients and calculate *P*-values using unpaired two-tailed Student's *t-*tests. All data for quantitative RT-PCR, Affy expression array values, immunofluorescence image quantification and Western blots are represented as mean ± s.e.m.

## Results

### SMAD activation in BRAF**^V600E^-**driven PTCs is regulated via MAPK

**Figure 1:**
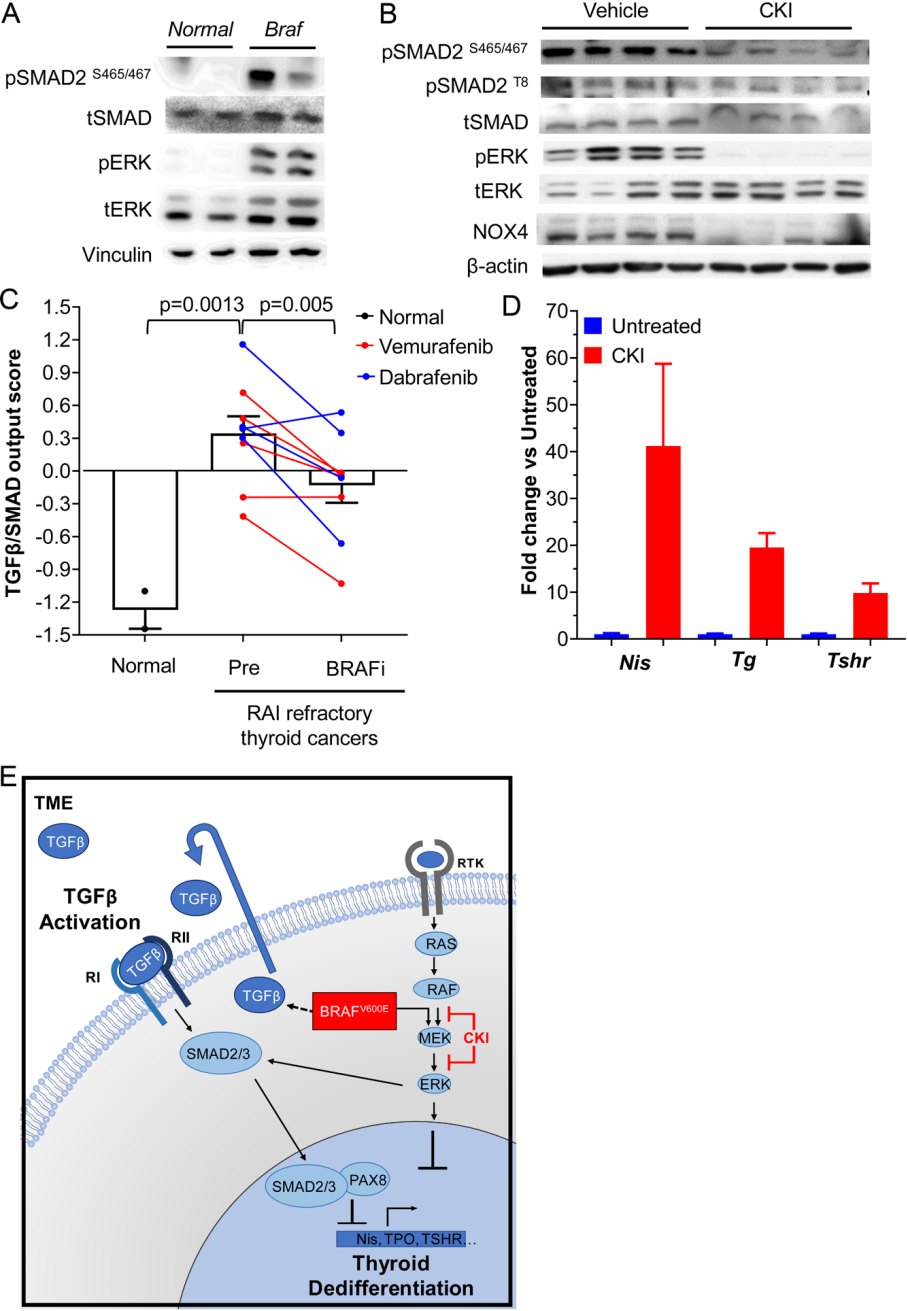
SMAD pathway activation in BRAF^V600E^-induced PTCs. (A) Western blot of normal thyroid and tumor lysates from *TPO-Cre/LSL-Braf^V600E^* (*Braf*) mice probed with antibodies to the indicated proteins. (B) Western blot of tumor tissues from *Braf* mice treated with vehicle or the MEK inhibitor CKI127 (CKI) probed for the indicated targets. Each lane corresponds to an individual mouse PTC (*n* = 4 per group). (C) TGFβ-SMAD transcriptional output scores of normal thyroid tissues compared to biopsy samples from patients with RAI-refractory thyroid cancers taken prior to and while on treatment with the RAF kinase inhibitors dabrafenib (blue) or vemurafenib (red) for 2 weeks. Each line depicts paired biopsy results from the same lesion prior to and while on drug. (D) Quantitative RT-PCR of thyroid differentiation markers in tumors from panel B. (E) Interactions between the MAPK pathway and TGFβ signaling in thyroid cancer. Oncogenic BRAF induces dedifferentiation in part by ERK-induced silencing or inactivation of lineage transcription factors and by interfering with TSH-induced cAMP signaling (Mitsutake* et al.* 2005) (not shown). BRAF^V600E^ also increases tumor cell secretion of TGFB1, leading to SMAD impairment of transactivation of thyroid-specific genes by the lineage transcription factor PAX8. Oncogenic BRAF also induces pERK phosphorylation at the T8 residue of SMAD, promoting its additional phosphorylation and activation by the TGFB1 receptor.

To determine whether the TGFβ/SMAD pathway is activated in BRAF^V600E^-driven PTC *in vivo,* we performed pSMAD Western blots in tumor lysates from TPO-Cre/LSL-Braf^V600E^ mice, which develop thyroid cancers with high penetrance by 5 weeks of age (Franco *et al.* 2011). As shown in Fig. 1A there was a marked increase in pSMAD in BRAF^V600E^-PTCs compared to normal thyroid. Treatment of mice with 1.5 mg/kg of the MEK inhibitor CH5126766 (CKI), which binds to MEK and places it in an inactive conformation bound to RAF (Ishii *et al.* 2013), potently blocked MAPK signaling and reduced pSMAD levels as well as expression of the TGFβ downstream target NOX4 (Fig. 1B) (Azouzi *et al.* 2017). MEK inhibition also reduced phospho-SMAD T8 levels (Fig. 1B), which has been previously shown to be required for optimal TGFβ signaling in mouse thyroid follicular cells (Knauf *et al.* 2011). In humans, BRAF^V600E^-driven PTCs had increased SMAD transcriptional output compared to normal thyroid, which was reduced by treatment with RAF inhibitors (Fig. 1C). As previously demonstrated, CKI increased the expression of genes involved in thyroid hormone biosynthesis (Fig. 1D) (Nagarajah *et al.* 2016). Hence, MAPK and pSMAD signaling are markedly increased in PTC driven by oncogenic BRAF, and the activity of both these pathways is MEK-dependent. Both could converge to impair the thyroid-differentiated function of the cancer cells, but their relative contribution to these effects has not been established (Fig. 1E).

### Role of TGFβ/SMAD in BRAF^V600E^-induced thyroid differentiation *in vivo*

**Figure 2:**
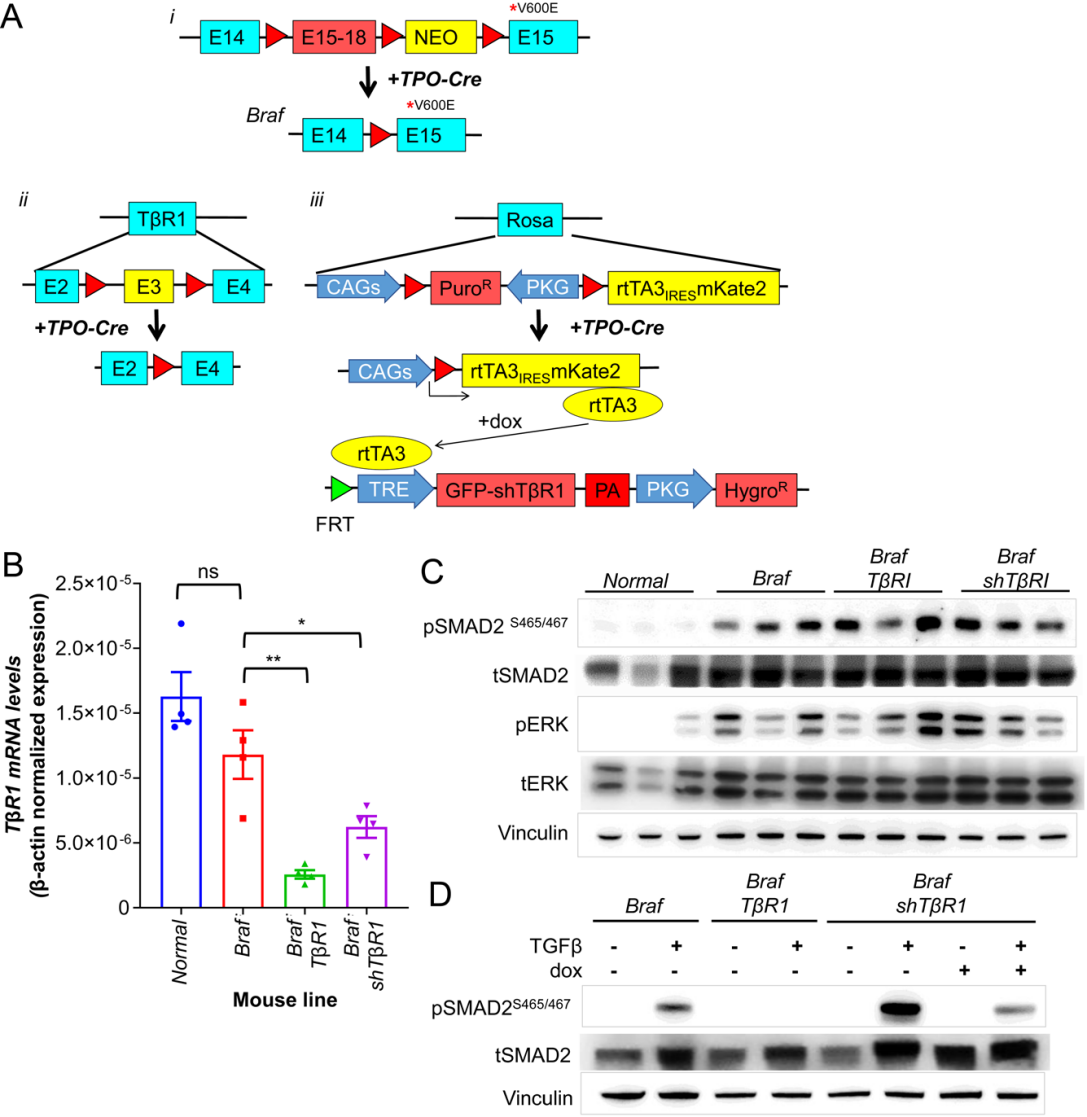
*TβR1* knockout in mouse BRAF^V600E^-PTCs is insufficient to inhibit SMAD activation* in vivo*. (A) Schematic design of the transgenic lines used in this study and of their recombination when crossed with *TPO-Cre* mice: (1) *Lsl-Braf^V600E^*; (2) *TβR1^f/f^* and (3) *tetO-shTβR1/RIK*. (B) β-actin normalized expression of *TβR1* mRNA in thyroid tissues of the indicated mouse lines as determined by quantitative RT-PCR (*n* = 4/group). *P < 0.05, **P < 0.005, ns = not significant. (C) Western blots for pSMAD and pERK in lysates from normal thyroid or PTCs from *Braf*, *Braf*/*TβR1* and *Braf/shTβR1* mice. (D) Western blots of cell lines derived from PTCs of *Braf*, *Braf/TβR1*, and *Braf/shTβR1* mice treated with vehicle or 1 ng/mL TGFβ1 for 1 h. Cells treated with dox were incubated for 3 days prior to collection.

To explore the role of TGFB1 on thyroid-differentiated gene expression in thyroid tumors, we first tested the TGFB1-neutralizing MAB 1D11 and the TGFBR1 (TβR1) kinase inhibitor SD-208 in cell lines derived from mouse BRAF^V600E^-PTCs, where both effectively blocked the TGFB1-induced SMAD2/3 phosphorylation (Supplementary Fig. 1A). For *in vivo* experiments, we chose to test the 1D11 antibody, because SD-208 has inhibitory effects on other kinases besides TβR1 (Tandon *et al.* 2015). Treatment of mice with a 1D11 schedule known to block the TGFB1 activity *in vivo* (Biswas *et al.* 2011) did not inhibit pSMAD in mouse BRAF^V600E^-PTCs (Supplementary Fig. 1B) or enhance the radioiodine uptake (Supplementary Fig. 1C). As this may have been due to the PK of the antibody in the tumor, we generated mouse models of BRAF^V600E^-PTC with thyroid-specific knockout of the *TgfβR1* gene, *TPO-Cre/LSL-Braf^V600E^/TβR1^fl/fl^* (*Braf/TβR1*) or, to avoid confounding effects of loss of the receptor during development, through post-natal dox-inducible expression of a *TβR1* shRNA. We validated a series of *TβR1* hairpins *in vitro*, and selected *TβR1* shRNA#6 to generate the mouse model *TPO-Cre/LSL-Braf^V600E^/TetO-GFP_shTβR1/RIK* (*Braf/shTβR1*) (Fig. 2A and Supplementary Fig. 1D, F). The penetrance and histological characteristics of the PTCs in *Braf/TβR1 and Braf/shTβR1* was indistinguishable from those of *Braf* mice (not shown). Despite appropriate knockdown of TβR1 with either model (Fig. 2B), pSMAD levels remained high in thyroid cancer tissues (Fig. 2C), and there was no significant increase in expression of thyroid differentiation markers (Supplementary Fig. 1G). Using cell lines derived from PTCs of *Braf/shTβR1* and *Braf/TβR1* mice, we found that the loss of* TβR1* effectively blocked TGFB1-induced SMAD activation *in vitro* (Fig. 2D). We concluded that Smad activation in this *in vivo* context was at least in part TβR1-independent.

### Activins induce SMAD in BRAF^V600E^-induced thyroid cancers

**Figure 3:**
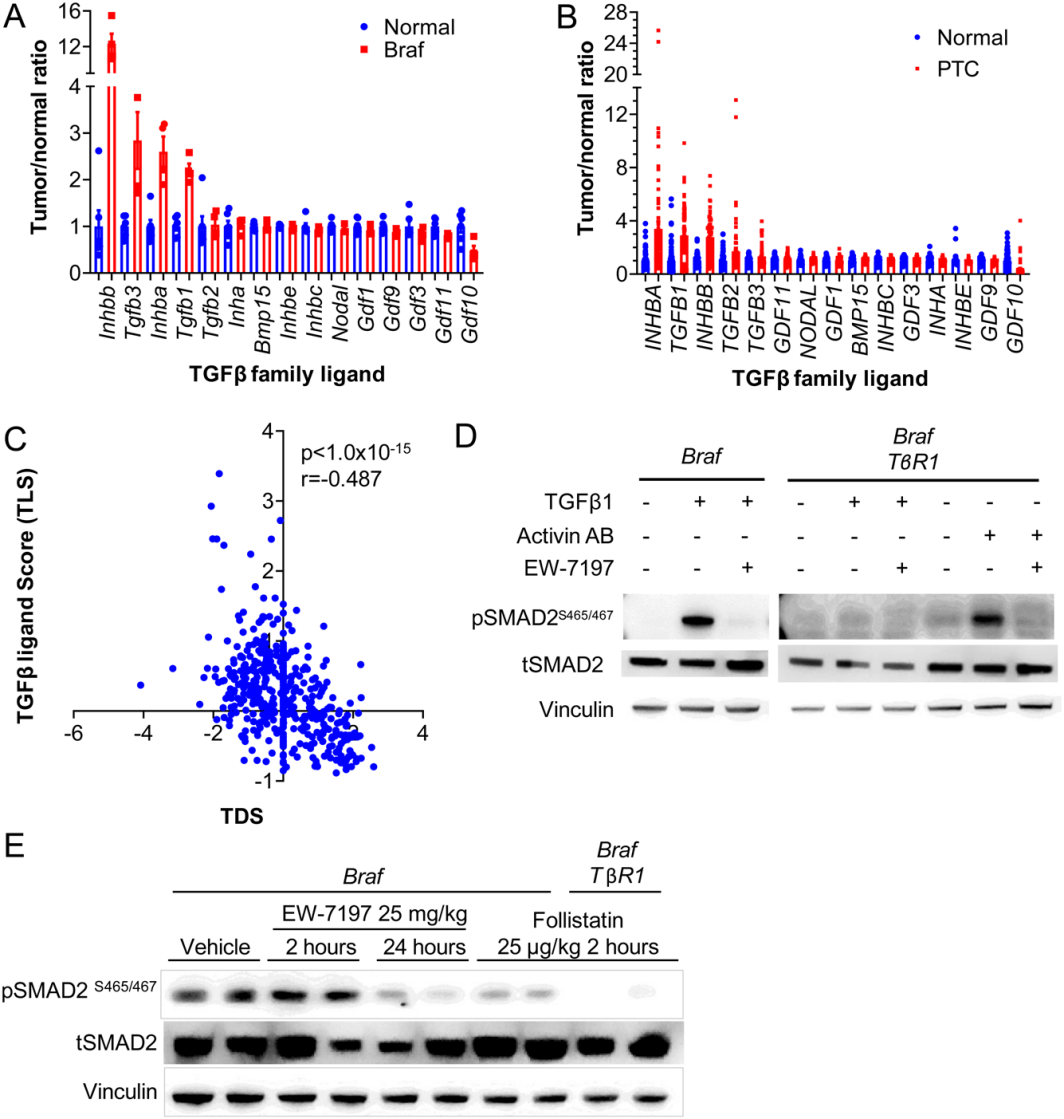
Blocking both ALK4 and ALK5 is required to completely inhibit SMAD activation in Braf^V600E^-induced PTCs. (A and B) Expression of the Tgfβ family ligands from Affymetrix expression arrays in (A) mouse and (B) human normal thyroids and *PTCs*. (C) Pearson correlation coefficient between the integrated TGFβ family ligand score (TLS) and TDS using the TCGA PTC dataset (Cancer Genome Atlas Research Network 2014). (D) Western blots of thyroid tumor lysates from *Braf* mice treated with EW7197 and of *Braf/TβR1* mice treated with follistatin.

The lack of SMAD inhibition by blocking TGFB1 or TGFBR1 *in vivo* suggested that other ligands of the TGFβ family might be engaging in this pathway. Activins are disulfide-linked homodimers or heterodimers of primarily two β-subunits, βA and βB, which are transcribed from the *Inhba* and *Inhbb* genes, respectively. They activate SMAD2/3 signaling via the activin type 1 receptor ALK4. Expression array data from normal mouse thyroid and BRAF^V600E^-PTCs showed *Inhbb* expression to be increased by >15-fold, as compared to a <3-fold increase in *Tgfb1* (Fig. 3A). On average human PTCs have a three- to four-fold upregulation of the TGFβ family ligand mRNAs, with *INHBA*, *TGFB1* and *INHBB* being the most prominent (Fig. 3B and Supplementary Fig. 2A). Analysis of the TCGA dataset of PTC found an inverse correlation between the expression of either *INHBA*, *INHBB*, *TGFB1*, *TGFB2* or *TGFB3* with the thyroid differentiation score (TDS). The reciprocal relationship with TDS was even stronger when compared with an integrated TGFβ ligand score encompassing expression levels of all five TGFβ family members (Fig. 3C).

Treatment of cell lines derived from *Braf* and *Braf/TβR1* mouse PTCs with activin AB markedly induced SMAD phosphorylation. Hence, SMAD activation in BRAF^V600E^-PTC cells can be induced via *TβR1* (*Alk5*) or *Acvr1b* (*Alk4*) receptors, which is blocked by the dual ALK4 and ALK5 inhibitor EW7197 (vactosertib) (Fig. 3D).

To investigate the roles of activin and TGFB1 on SMAD activation *in vivo*, tumor-bearing *Braf* and *Braf/TβR1* mice were treated with EW7197 or with follistatin, a potent inhibitor of several TGFβ family ligands (primarily activin and to a lesser extent BMPs, myostatin, and possibly TGFB3 – but not TGFB1 or 2) (Nogai *et al.* 2008, Sepporta *et al.* 2013). Treatment of *Braf* mice with EW7197 or follistatin effectively decreased pSMAD in the PTCs. Follistatin had a greater inhibitory effect on SMAD phosphorylation in PTCs from *Braf/TβR1* as compared to *Braf* mice, indicating that both activin and Tgfβ co-regulate this pathway in these tumors (Fig. 3E).

**Figure 4:**
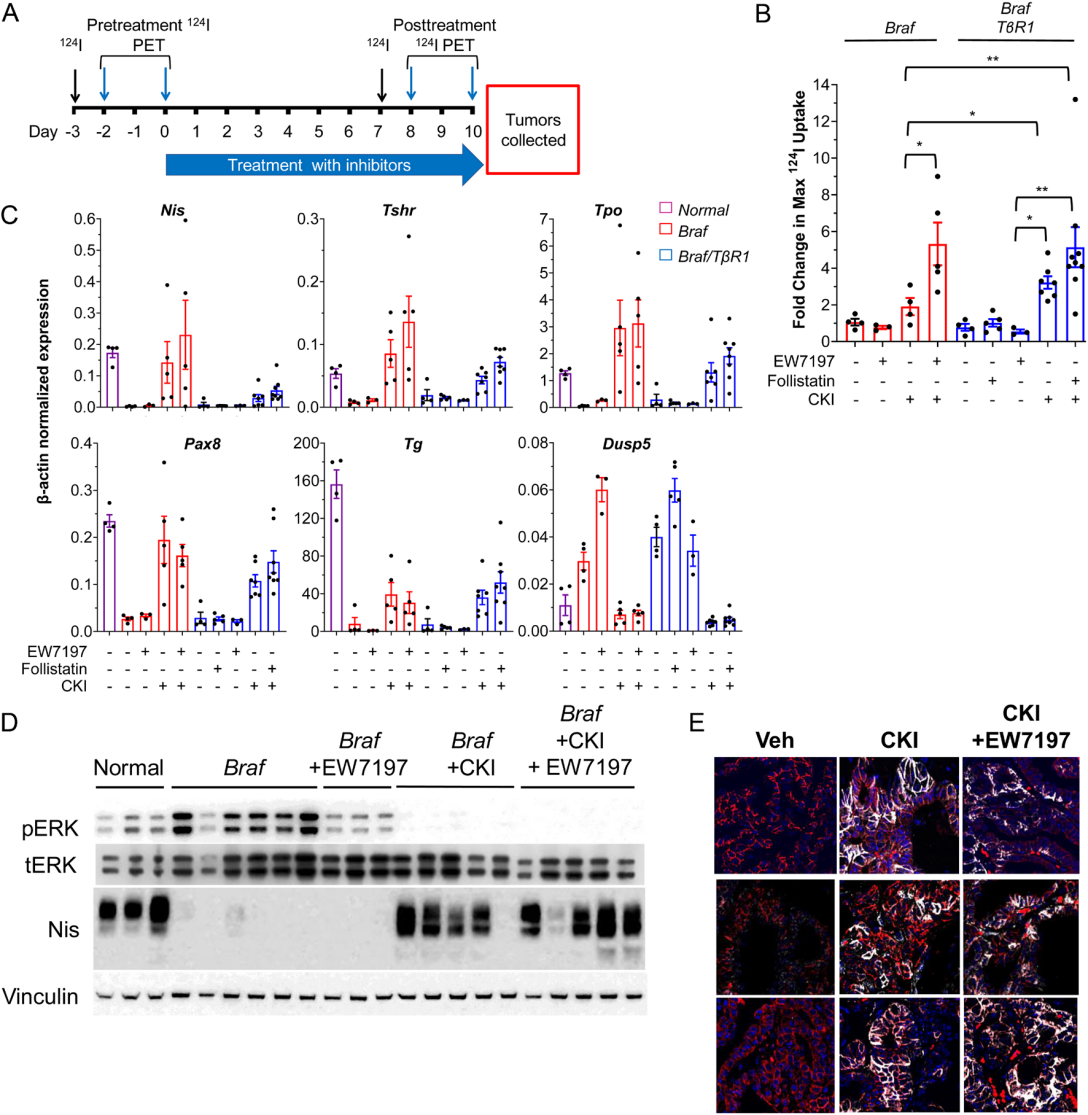
Co-inhibition of ALK4 and 5 in combination with MAPK blockade increases ^124^I uptake in *Braf* mice. (A) Schema for mouse experiments testing effects of CKI and/or EW7197 or follistatin on ^124^I incorporation into thyroid tumors from the indicated mouse models. (B) Fold-change in maximum ^124^I uptake in *Braf* and *Braf/TβR1* mice treated with CKI, EW7197 or follistatin or the indicated combinations (*P < 0.05, **P < 0.01). (C and D) Quantitative RT-PCR (C) and Western blots (D) of tumor tissues from B (collected after second ^124^I PET study). (E) Immunofluorescent staining for NIS and pan-cadherin in tumors from mice treated as indicated in (A).

To explore the role of SMAD in BRAF^V600E^-induced loss of iodide uptake in PTCs, *Braf* mice were treated with a 10 days course of EW7197 or follistatin. Quantitative ^124^I-PET scans were performed prior to therapy and at days 7–10 (Schema in Fig. 4A). EW7197 or follistatin monotherapy had no effect on ^124^I uptake in PTCs of either *Braf* or* Braf/TβR1* mice. Accordingly, these drugs failed to rescue expression of *Nis* or any other thyroid differentiation marker in PTCs from either mouse model (Fig. 4).

We next examined whether SMAD inhibition further increased the iodide uptake induced by CKI. ^124^I PET imaging was performed prior to and while on treatment with CKI alone or in combination with the SMAD pathway inhibitors (Fig. 4A). Treatment with CKI caused a greater increase in ^124^I uptake in *Braf/TβR1* compared to *Braf* mice (Fig. 4B). EW7197, which inhibits pSMAD profoundly in BRAF^V600E^-PTCs (Fig. 3C), also augmented the effects of CKI in *Braf* mice. Accordingly, there was a trend toward higher iodine uptake in *Braf/TβR1* mice treated with the combination of follistatin and CKI compared to CKI alone. Taken together, combined blockade of ALK4 and ALK5 by pharmacological targeting alone (EW7197) or in combination with genetic ablation (follistatin + *TβR1* knockout) cooperates with MAPK inhibition to augment iodine uptake in Braf-mutant PTC.

Potent inhibition of pSMAD with either EW7197 in *Braf* mice or follistatin in *Braf/TβR1* mice did not increase *Nis*, *Tpo*, *Tg*, or *Pax8* mRNA levels, consistent with their lack of effect on iodide uptake (Fig. 4C). By contrast, treatment with CKI markedly increased all four markers. When CKI was combined with EW7197, there was a trend toward higher *Nis* and *Tshr* mRNA. CKI restored NIS protein to levels approaching those seen in normal thyroid, but the addition of EW7197 did not result in further augmentation (Fig. 4D and Supplementary Fig. 3A). TGFβ has been shown to block the recruitment of immune cells to the tumor microenvironment (Tauriello *et al.* 2018). Treatment of *Braf* mice with EW7197 or CKI + EW7197 did not increase CD45+ cell infiltration (Supplementary Fig. 3B), indicating that decreased tumor cell purity was unlikely to account for the lack of clear increase in thyroid differentiation markers in the tissue extracts. SMAD activation has been shown to reduce plasma membrane localization of NIS via upregulation of NOX4 (Azouzi *et al.* 2017). We tested this by IHC for NIS in tumors from *Braf* mice treated with vehicle, CKI or CKI + EW7197. CKI markedly increased membrane-localized NIS compared to vehicle, which was not further augmented with the CKI + EW7197 combination (Fig. 4E and Supplementary Fig. 3C).

## Discussion

PTCs driven by BRAF^V600E^ have a greater frequency of nodal recurrences and are usually refractory to RAI treatment (Nikiforova *et al.* 2003, Elisei *et al.* 2008, Xing *et al.* 2013). They are also overrepresented in FDG-PET positive metastatic disease (Ricarte-Filho *et al.* 2009). The relative resistance to ^131^I is explained by the disruption in expression of NIS and other thyroid-specific genes that are required for the incorporation of iodine into thyroid cells (1;2). Multiple mechanisms that are not mutually exclusive have been proposed to explain these effects: (1) BRAF activation inhibits PAX8 expression, a paired domain lineage transcription factor that regulates transcription of *NIS* and other thyroid-specific genes. (2) NKX2-1, a transcription factor essential for thyroid-differentiated gene expression is phosphorylated by ERK, which disrupts its ability to transactivate target genes (Missero *et al.* 2000). (3) Expression of BRAF^V600E^ increases TGFB1 secretion in rat thyroid PCCL3 cells creating an autocrine loop that activates SMADs (Riesco-Eizaguirre *et al.* 2009), which in turn bind to PAX8 and impair its transactivation of the *Nis* gene promoter (Costamagna *et al.* 2004). This induction of the TGFB1 autocrine loop by BRAF^V600E^ in PCCL3 cells was shown to be MEK-independent (Riesco-Eizaguirre *et al.* 2009).

The objective of this study was to determine the role of the TGFβ signaling pathway on thyroid differentiation in genetically accurate models of BRAF^V600E^-induced thyroid cancer *in vivo*. We found that SMAD phosphorylation was induced in mouse BRAF^V600E^-PTCs, and that a TGFβ transcriptional output signature was present in advanced RAI-refractory human BRAF-mutant thyroid cancers. We employed pharmacological and genetic approaches to target TGFβ signaling in the mouse models and found that either approach effectively blocked TGFβ signaling in cell lines derived from the murine BRAF^V600^-PTCs but failed to do so *in vivo*. We found that other ligands in the TGFβ family were activating SMAD *in vivo*, which was buttressed by evidence of marked upregulation of Inhbb in BRAF^V600E^-PTCs. Accordingly, SMAD phosphorylation was inhibited in the PTCs by follistatin, an activin inhibitor. However, follistatin was only able to completely block pSMAD activation in mice with thyrocyte-specific genetic ablation of TβR1, indicating that both activin and TGFβ play a role in SMAD activation, with activin being dominant. We, therefore, chose the ALK4/5 inhibitor EW7197 to explore the role of SMAD activation on iodide uptake in mouse BRAF^V600E^-driven PTCs, leading to the conclusion that potent inhibition of SMAD alone was insufficient to restore expression of genes involved in thyroid hormone production or iodide uptake.

We previously showed that both BRAF^V600E^-PTC cells and tumor-associated macrophages contribute TGFB1 to the tumor microenvironment (Knauf *et al.* 2011). RNAseq of flow-sorted PTC cells from the *TPO-Cre/LsL-Braf^V600E^* model used in this paper showed that *Tgfb1* and Activin mRNA levels are higher in the BRAF^V600E^-PTC cells compared to normal thyroid follicular cells (not shown). We do not know which cell types, other than BRAF^V600E^-PTC cells, contribute to the intratumoral pool of activin.

Potent MAPK pathway blockade promotes redifferentiation and increased iodide uptake in BRAF^V600E^-driven thyroid cancer (Nagarajah *et al.* 2016). Here, we show that this is associated with a decrease in SMAD activation, which is likely due to a reduction in ERK-dependent phosphorylation of the threonine 8 residue of SMAD. Phosphorylation at this site by MAPK amplifies the activation of SMAD by ligand-bound TGFβ family receptors (Wrighton *et al.* 2009, Knauf *et al.* 2011). These convergent effects on SMAD may explain the increase in iodide uptake in Braf mice treated with the combination of EW7197 and CKI compared to CKI alone. However, MAPK activation suppresses the differentiation program largely through SMAD-independent mechanisms, since potent inhibition of SMAD failed to restore thyroid-differentiated gene expression in the setting of persistent MAPK pathway activation. This likely explains why the treatment combination only resulted in a trend toward increased *Nis* and *Tshr* mRNA compared to MEK inhibition alone.

In summary, activation of the MAPK pathway by BRAF^V600E^ promotes SMAD activation *in vivo* through TGFβ and activin-induced pathways. Increased expression of TGFβ and activin ligands is common in PTCs and this is inversely correlated with an integrated transcriptional output score associated with thyroid differentiation. TGFβ-induced signaling through SMAD has been shown to suppress the expression of NIS and other thyroid differentiation markers in BRAF-WT thyroid cells (Colletta *et al.* 1989, Nicolussi *et al.* 2003, Costamagna *et al.* 2004). However, signaling via SMAD is not the primary mechanism for suppression of thyroid-specific gene expression or loss of iodide uptake in the context of MAPK activation *in vivo*, as inhibition of SMAD alone did not improve any of these biomarkers. The precise mechanism underpinning the additive effect of SMAD and MEK inhibition on iodide uptake is unclear. It was not associated with clear differences in the expression of NIS. NIS abundance and membrane localization are only some of the variables that impact iodide incorporation (others include iodide organification and iodide efflux, which we did not assess).

The enhancement of iodine uptake may reflect the close interplay between these two pathways, which mutually reinforce overlapping effects on the thyroid-differentiated state during dynamic changes in their respective signaling outputs. The translational implication of these findings is intriguing and has not been explored so far. Any clinical trials looking into synergistic effects of blocking SMAD and MAPK pathways should select agents that target both ALK4 and ALK5 and consider using them for short intervals because of the potential tumor-promoting effects of SMAD inhibition in cancer.

## Supplementary Material

Supplemental Table 1: List of primers used.

Supp Figure 1: Targeting Tgfβsignaling in BRAFV600E-induced PTCs. A) Western blots of protein lysates from a cell line derived from a BrafV600EPTC incubated in serum free medium for 24h and then treated with TGFβ1 for 1 h in the absence or presence of the TGFβ1 blocking antibody 1D11 or the TGFβR1 kinase inhibitor SD208 (1 μM). B, C)Effect of TGFβ1 blockade on SMAD activation and 124I incorporation in vivo. Brafmice were treated with isotypecontrol IgGor 1D11 antibody once every 2d for 14d. B) Western blots of PTC lysates collected at day 14 probed with the indicated antibodies. C)Thyroid 124I uptake (%ID/g ±SEM) in Brafmice before and after treatment with 1D11 or isotypecontrol. 124I uptake was measured by micro-PET 24 h post 124I administration before and after 14d of treatment. Each cohort contained 4 mice. D,E) Knockdown efficiency screen shRNAsto TgfβR1 (D)and TgfβR2 (E)in mouse Braf-PTC cells. Top:Quantitative RT-PCR performed in triplicate. Bottom:Western blots for pSMADof TGFβ1 treated cells. F) Comparison of TgfβR1and TgfβR2shRNAson TGFβ1-induced SMAD activation in Braf-PTC cells. G) Quantitative RT-PCR of thyroid differentiation markers in PTCs from Braf, Braf/TβR1and Braf/shTβR1mice. Four mouse thyroid tissues from each genotype were analyzed in triplicate. Unpaired t test with Welch’s correction: *p<0.05, ** p<0.01 compared to Brafmice.

Supp Figure 2: TGFβfamily ligand expression is anticorrelatedwith thyroid differentiation score (TDS) in PTC.A) Heat map of TGFβfamily ligand mRNA expression in human PTCs compared to normal thyroid tissues. Fold-change in mRNAs was calculated from public transcriptomicdata (GSE29265, GSE33630, GSE65144). B) Pearson correlation coefficient between expression of the indicated TGFβ ligands and TDS using the TCGA PTC dataset (Network CGAR (2014). Integrated genomic characterization of papillary thyroid carcinoma. Cell159:676-690).

Supp Figure 3: Quantification of NIS protein levels in Western blots of CKI and EW7197-treated Brafmice.A) Bars represent the mean vinculinnormalized expression of NIS ±SEM from the Western blots shown in Fig 4E. B) Immunohistochemistry for CD45 in PTCs from Brafmice treated with vehicle, CKI or CKI+EW7197. C) Membrane expression of NIS (mean ±SEM) from co-immunofluorescence staining in Fig 4F. *p<0.05, ** p<0.002

## Declaration of interest

JAF is a consultant for Loxo Oncology, received grant support from Eisai and is a co-inventor of intellectual property focused on HRAS as a biomarker for treating cancer using tipifarnib which has been licensed by MSK to Kura.

## Funding

This work was supported by grants from the US National Institutes of Health http://dx.doi.org/10.13039/100000002 RO1-CA50706-23 (JAF), R01-CA 249663-01A1 (JAF, ALH), RO1-CA184724-01A1 (ALH, JAF), P50-CA 172012-01 (JAF) and P30-CA008748/CA/NCI (Craig Thompson http://dx.doi.org/10.13039/100004686 PI), Cycle for Survival (ALH), the Jayme and Peter Flowers fund, the Frank D Cohen fund, Cycle for Survival and the Translational Research Oncology Training program 5T32CA160001 (MS).
