# Supplementary material for: Co-inhibition of SMAD and MAPK signaling enhances 124I uptake in BRAF-mutant thyroid cancers
Source: Endocr Relat Cancer. 2021 Apr 23;28(6):391–402. doi: 10.1530/ERC-21-0017 (PMC8183640; doi:10.1530/ERC-21-0017)
Supplement: Supp Figure 1: Targeting Tgfβsignaling in BRAFV600E-induced PTCs. A) Western blots of protein lysates from a cell line derived from a BrafV600EPTC incubated in serum free medium for 24h and then treated with TGFβ1 for 1 h in the absence or presence of the TGFβ1 blocking antibody 1D11 or the TGFβR1 k [file supplementary_figure_1.pdf]

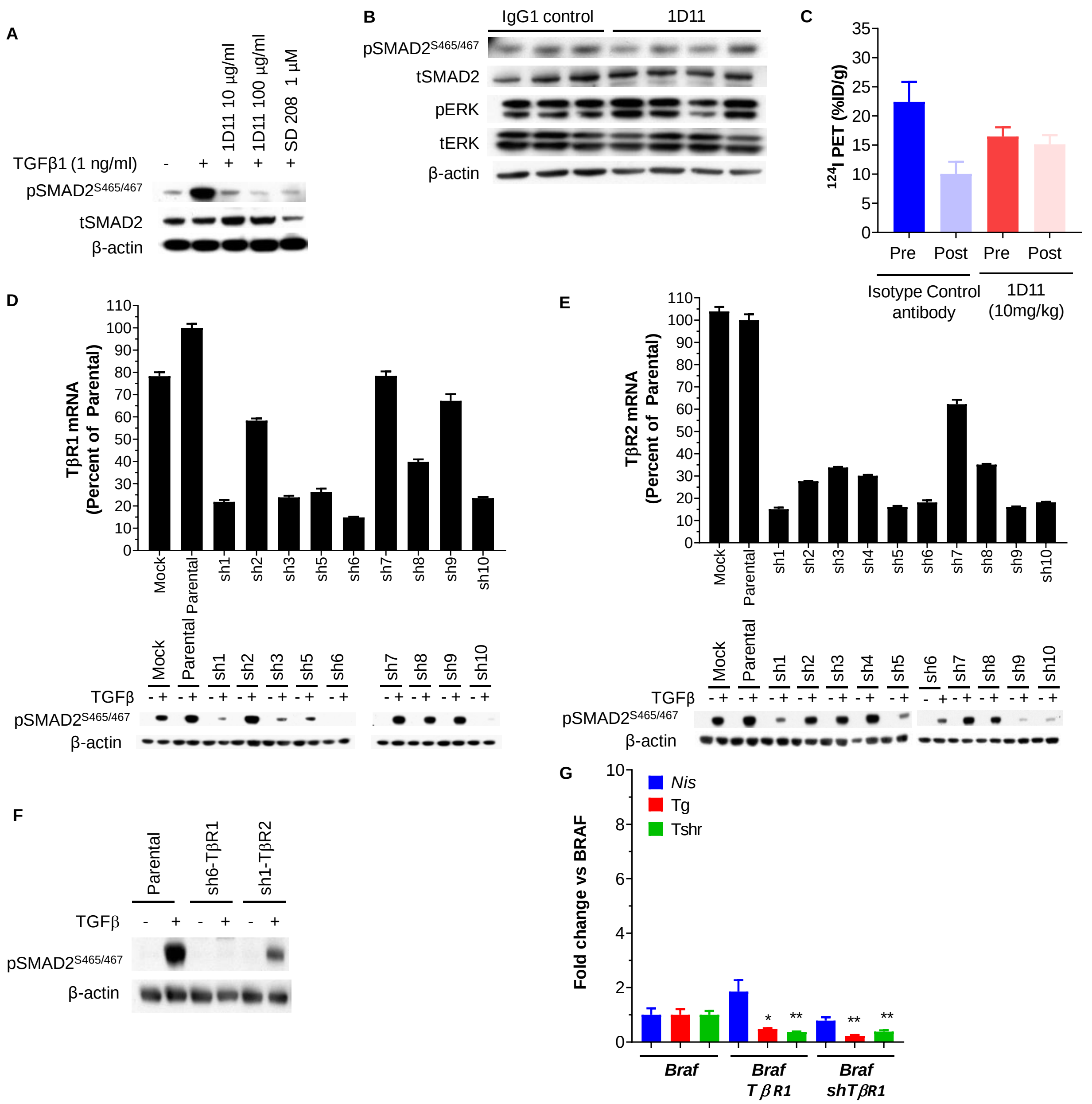

Supp Figure 1: Targeting *Tgfβ signaling in BRAF^{V600E}-induced PTCs.* **A**) Western blots of protein lysates from a cell line derived from a Braf^{V600E} PTC incubated in serum free medium for 24h and then treated with TGFβ1 for 1 h in the absence or presence of the TGFβ1 blocking antibody 1D11 or the TGFβR1 kinase inhibitor SD208 (1 μM). **B, C)** Effect of TGFβ1 blockade on SMAD activation and ^{124}I incorporation *in vivo*. Braf mice were treated with isotype control IgG or 1D11 antibody once every 2d for 14d. **B**) Western blots of PTC lysates collected at day 14 probed with the indicated antibodies. **C)** Thyroid ^{124}I uptake (%ID/g ±SEM) in *Braf* mice before and after treatment with 1D11 or isotype control. ^{124}I uptake was measured by micro-PET 24 h post ^{124}I administration before and after 14d of treatment. Each cohort contained 4 mice. **D,E**) Knockdown efficiency screen shRNAs to TgfβR1 **(D)** and TgfβR2 **(E)** in mouse Braf-PTC cells. *Top:* Quantitative RT-PCR performed in triplicate. *Bottom:* Western blots for pSMAD of TGFβ1 treated cells. **F**) Comparison of *TgfβR1* and *TgfβR2* shRNAs on TGFβ1-induced SMAD activation in Braf-PTC cells. **G**) Quantitative RT-PCR of thyroid differentiation markers in PTCs from *Braf*, *Braf/TβR1* and *Braf/shTβR1* mice. Four mouse thyroid tissues from each genotype were analyzed in triplicate. Unpaired t test with Welch's correction: *p<0.05, ** p<0.01 compared to *Braf* mice.
