# Supplementary material for: Co-inhibition of SMAD and MAPK signaling enhances 124I uptake in BRAF-mutant thyroid cancers
Source: Endocr Relat Cancer. 2021 Apr 23;28(6):391–402. doi: 10.1530/ERC-21-0017 (PMC8183640; doi:10.1530/ERC-21-0017)
Supplement: Supp Figure 2: TGFβfamily ligand expression is anticorrelatedwith thyroid differentiation score (TDS) in PTC.A) Heat map of TGFβfamily ligand mRNA expression in human PTCs compared to normal thyroid tissues. Fold-change in mRNAs was calculated from public transcriptomicdata (GSE29265, GSE33630, GSE6 [file supplementary_figure_2.pdf]

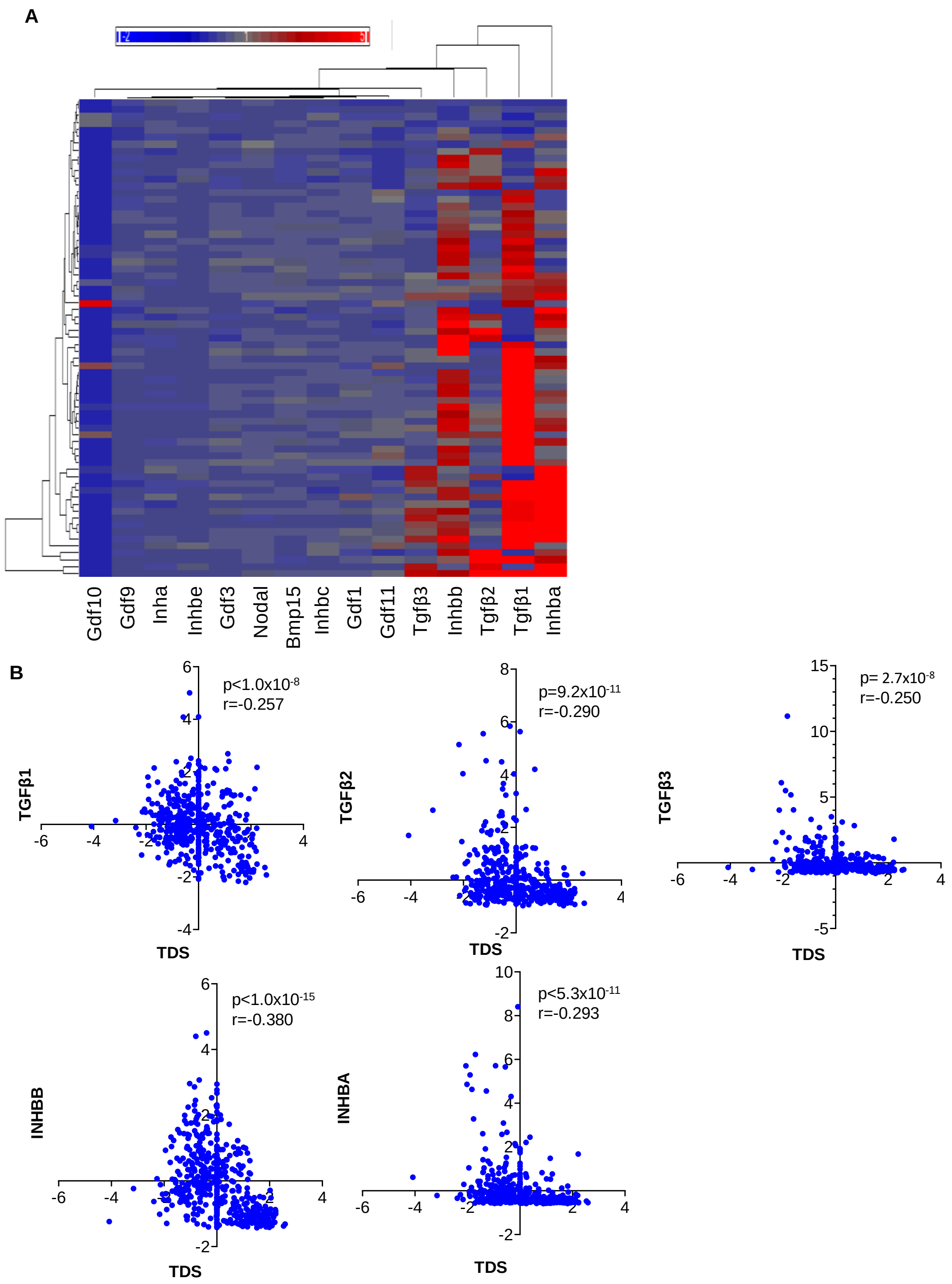


**Supp Figure 2**: *TGFβ family ligand expression is anticorrelated with thyroid differentiation score (TDS) in PTC.* **A**) Heat map of TGFβ family ligand mRNA expression in human PTCs compared to normal thyroid tissues. Fold-change in mRNAs was calculated from public transcriptomic data (GSE29265, GSE33630, GSE65144). **B**) Pearson correlation coefficient between expression of the indicated TGFβ ligands and TDS using the TCGA PTC dataset (Network CGAR (2014). Integrated genomic characterization of papillary thyroid carcinoma. *Cell* **159:** 676-690).
