# Supplementary material for: Co-inhibition of SMAD and MAPK signaling enhances 124I uptake in BRAF-mutant thyroid cancers
Source: Endocr Relat Cancer. 2021 Apr 23;28(6):391–402. doi: 10.1530/ERC-21-0017 (PMC8183640; doi:10.1530/ERC-21-0017)
Supplement: Supp Figure 3: Quantification of NIS protein levels in Western blots of CKI and EW7197-treated Brafmice.A) Bars represent the mean vinculinnormalized expression of NIS ±SEM from the Western blots shown in Fig 4E. B) Immunohistochemistry for CD45 in PTCs from Brafmice treated with vehicle, CKI or CKI [file supplementary_figure_3.pdf]

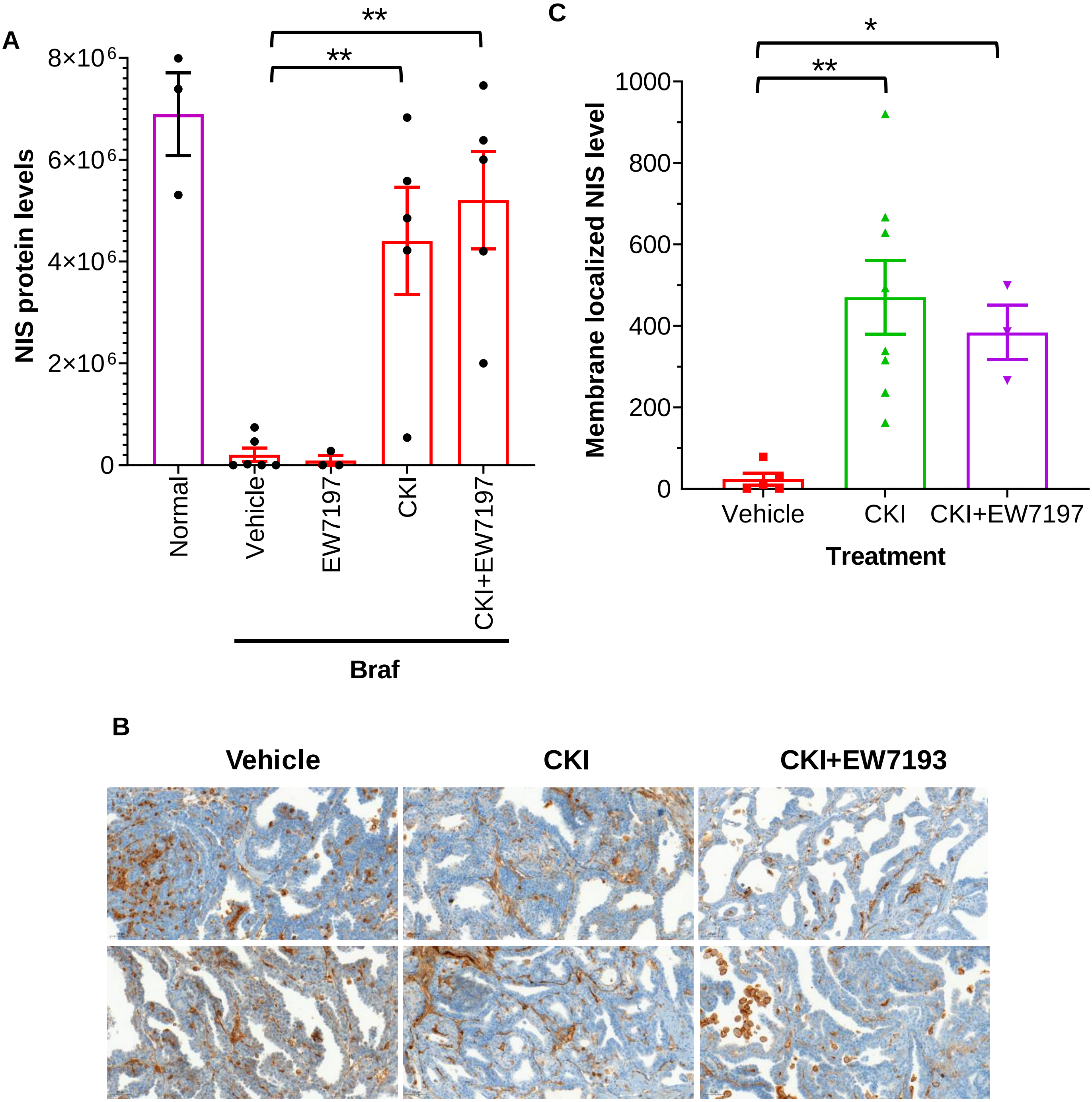


**Supp Figure 3**: *Quantification of NIS protein levels in Western blots of CKI and EW7197-treated Braf mice.* **A**) Bars represent the mean vinculin normalized expression of NIS ±SEM from the Western blots shown in Fig 4E. **B**) Immunohistochemistry for CD45 in PTCs from Braf mice treated with vehicle, CKI or CKI+EW7197. **C**) Membrane expression of NIS (mean ±SEM) from co-immunofluorescence staining in Fig 4F. *p<0.05, ** p<0.002
